# Influence of oral premedication and prewarming on core temperature of cardiac surgical patients: a prospective, randomized, controlled trial

**DOI:** 10.1186/s12871-019-0725-7

**Published:** 2019-04-12

**Authors:** Anselm Bräuer, Michaela Maria Müller, Anna Julienne Wetz, Michael Quintel, Ivo Florian Brandes

**Affiliations:** Department of Anaesthesiology, University Medical Center Göttingen, Robert-Koch-Str. 40, 37075 Göttingen, Germany

**Keywords:** Premedication, Benzodiazepine, Hypothermia, Prewarming, Forced-air warming

## Abstract

**Background:**

Perioperative hypothermia is still very common and associated with numerous adverse effects. The effects of benzodiazepines, administered as premedication, on thermoregulation have been studied with conflicting results. We investigated the hypotheses that premedication with flunitrazepam would lower the preoperative core temperature and that prewarming could attenuate this effect.

**Methods:**

After approval by the local research ethics committee 50 adult cardiac surgical patients were included in this prospective, randomized, controlled, single-centre study with two parallel groups in a university hospital setting. Core temperature was measured using a continuous, non-invasive zero-heat flux thermometer from 30 min before administration of the oral premedication until beginning of surgery. An equal number of patients was randomly allocated via a computer-generated list assigning them to either prewarming or control group using the sealed envelope method for blinding. The intervention itself could not be blinded. In the prewarming group patients received active prewarming using an underbody forced-air warming blanket. The data were analysed using Student’s t-test, Mann-Whitney U-test and Fisher’s exact test.

**Results:**

Of the randomized 25 patients per group 24 patients per group could be analysed. Initial core temperature was 36.7 ± 0.2 °C and dropped significantly after oral premedication to 36.5 ± 0.3 °C when the patients were leaving the ward and to 36.4 ± 0.3 °C before induction of anaesthesia. The patients of the prewarming group had a significantly higher core temperature at the beginning of surgery (35.8 ± 0.4 °C vs. 35.5 ± 0.5 °C, *p* = 0.027), although core temperature at induction of anaesthesia was comparable. Despite prewarming, core temperature did not reach baseline level prior to premedication (36.7 ± 0.2 °C).

**Conclusions:**

Oral premedication with benzodiazepines on the ward lowered core temperature significantly at arrival in the operating room. This drop in core temperature cannot be offset by a short period of active prewarming.

**Trial registration:**

This trial was prospectively registered with the German registry of clinical trials under the trial number DRKS00005790 on 20th February 2014.

## Background

Perioperative hypothermia, defined as core temperature < 36 °C, is still very common [1, 2]. Many well conducted prospective randomized trials [3–6] and large retrospective studies [7, 8] documented numerous adverse events associated with it. In the last decades many studies have focused on intraoperative prevention of perioperative hypothermia and recently prewarming is getting more attention [9, 10].

Benzodiazepines influence behavioural and autonomic thermoregulation by binding to GABA receptors in the brain. The effects of benzodiazepines on perioperative thermoregulation have been studied with conflicting results. In an early study Kurz et al. [11] found that even very high doses of midazolam had only moderate effects on core temperature and the vasoconstriction threshold of healthy volunteers. In contrast, Matsukawa found a clear dose dependent effect of midazolam on core temperature with a drop in core temperature of more than 0.5 °C when 0.075 mg.kg^− 1^ midazolam were administered. In another study the effects of midazolam on core temperature could be minimized by prewarming [12], which was started directly after the administration of midazolam. In Germany many patients get oral premedication with a benzodiazepine on the ward before being transported through the cold hospital corridors to the preoperative holding area or the operating room.

In this study we analysed whether the oral administration of a benzodiazepine has an influence on core temperature and if prewarming could attenuate this effect. The first hypothesis was that premedication with flunitrazepam would lower the core temperature significantly. The second hypothesis was that prewarming with forced-air would prevent a further drop of core temperature after induction of anaesthesia.

## Methods

After approval by the local Institutional Ethics Committee (Ethikkommission Universitätsmedizin Göttingen on 10^th^ of February 2014 under the number 16/12/13), and trial registration under German Clinical Trials Register (DRKS.de, Registration Trial DRKS00005790 on 20^th^ of February 2014) patient recruitment was started. Sample-size was estimated because no reference data was available to base a sample size calculation on. Afterwards, a power analysis was done to determine the power of the data. Between September 2014 and July 2016 50 patients were included in this prospective, randomised, controlled, single-centre study with two parallel groups. Written informed consent was obtained from all patients at least on the day prior to anaesthesia and surgery.

We included adult patients between 50 and 75 years with American Society of Anesthesiology (ASA) physical status ≤III and a body mass index (BMI) between 20 and 30 kg.m^− 2^. After premedication with 1 mg flunitrazepam, patients underwent elective cardiac surgery with cardiopulmonary bypass (CPB) at balanced anaesthesia using midazolam, sufentanil, rocuronium and sevoflurane. Exclusion criteria were: preoperative fever, a core temperature of less than 35 °C, a clinical relevant thyroid disease, a BMI > 30 kg.m^− 2^, or participation in another clinical trial.

### Randomisation

Patients were identified through the daily surgical schedule. A computer generated randomisation list (www.randomization.com seed 18,241) was used to allocate patients to one of the two study groups with an allocation ratio of 1:1. Patient randomisation was done after enrollment in the study by a member of the study team (MMM), and the sealed envelope method was used for blinding. The intervention itself (no prewarming or prewarming before induction of anaesthesia) could not be blinded.

### Measurements

In all patients core temperature was measured using a single use, continuous, non-invasive zero-heat flux thermometer (3 M™ SpotOn™ Temperature Monitoring System, 3 M, St. Paul, MN, USA) [13, 14] attached to the lateral forehead of the patients 30 min before administration of the oral premedication. After an equilibration period of a few minutes the device produces a skin surface temperature that is equivalent to the patient’ s deep tissue temperature by heating the skin beneath the sensor and by insulating the skin at the same time. Thereby the thermometer creates a so-called small isothermal tunnel of tissue in which almost no heat transfer to the environment occurs. Then the measured skin temperature should be equal to core temperature. To ensure continuous correct measurement the system was connected to a self-assembled power pack.

### Protocol

Patients in the control group without prewarming (control group) were insulated preoperatively with a hospital duvet on the ward and this insulation was used until the beginning of washing and draping. Patients of the treatment group with prewarming (prewarming group) were also insulated preoperatively with a hospital duvet. In addition, active prewarming using an underbody forced-air warming blanket (UNIVERSAL II, Moeck & Moeck, Hamburg, Germany) was started after arrival of the patients in the induction room. We aimed at 10–20 min of prewarming prior induction of anaesthesia according to the German guidelines for the prevention of inadvertent hypothermia [15].

During the prewarming time we checked the patient’s identity and if written informed consent for the study, the anaesthesia, and the surgery was signed by the patient. Then the patient was prepared for induction of anaesthesia by getting i.v. access and starting routine monitoring with ECG, oxygen saturation, and invasive arterial blood pressure measurement in the radial artery. Thus prewarming did not prolong procedure times.

After induction of anaesthesia patients were transferred into the operating room. In the operating room warming therapy using the underbody forced-air warming blanket was used in both patient groups during surgical desinfection. Desinfection of the skin was done three times using Braunoderm® (B.Braun Melsungen AG, Melsungen, Germany) and an impact time of ten minutes was used.

The following parameters were documented:

- Biometric data (age, weight, height, sex)
- ASA-Classification
- Core temperature approximately 30 min before oral premedication with 1 mg flunitrazepam
- Core temperature after oral premedication when the patients were leaving the ward
- Core temperature before induction of anaesthesia
- Level of sedation at arrival in the induction room using the Ramsay Score [16] by the same observer (MMM)
- Core temperature at beginning of surgery

### Statistical analysis

The data were analysed with SigmaPlot for Windows 12.0, Build 12.2.0.45. (Systat Software, Inc., San Jose, CA, USA). Normal distribution was tested with the Shapiro-Wilk test. Normally distributed data were described by mean and standard deviation, non-parametric data by median and interquantil range. Categorical data were given as percentages. Student’s t-test, Mann-Whitney U-test and Fisher’s exact test were used to compare the two groups as appropriate. A *p*-value of less than 0.05 was considered statistically significant.

The first null hypothesis that the premedication with flunitrazepam does not change the core temperature was tested using One Way Repeated Measures Analysis of Variance (ANOVA) using the core temperatures of all included patients for the time points oral premedication, after oral premedication when the patients were leaving the ward, and before induction of anaesthesia. Post hoc pairwise multiple comparison testing was performed with the Holm-Sidak method.

The second null hypothesis that prewarming with forced-air would not make a difference compared to no prewarming at the beginning of surgery was tested with a two-tailed t-test. In addition, the incidence of hypothermia at the beginning of surgery was compared using the Fisher’s exact test.

## Results

**Fig. 1:**
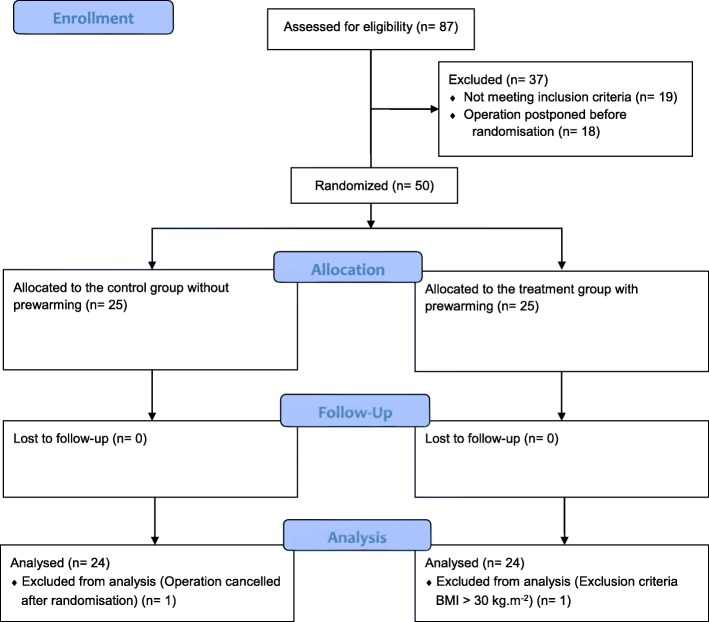
CONSORT diagram

After assessing 87 patients for eligibility 50 patients could be randomized into the two groups and 48 patients could be analysed. In each group one patient had to be excluded, because the surgery was cancelled after randomization in one patient and in another patient because of exclusion criteria (BMI > 30 kg.m^− 2^) (Fig. 1). We did a power analysis to estimate the power of the study with our chosen sample size. Using ANOVA with an alpha = 0.05, 24 patients in each group resulted in a power of 0.941. Using *t*-test with an alpha = 0.05, 24 patients in each group resulted in a power of 0.924.

The two patient groups were similar with respect to age, weight, sex, BMI, ASA-Classification, and type of the planned surgery (Table 1).

**Table 1:** Characteristics of patients receiving no prewarming (control group) and active prewarming with forced-air (prewarming group). Mean ± SD or Median and [IQR] as appropriate

Parameter	Control group	Treatment group	p-value
Age [yrs]	67.5 [62.25–72.0]	66.5 [60.25–71.0]	0.92
Sex [M/F]	15/9	20/4	0.19
Weight [kg]	75.6 ± 13.2	82.4 ± 10.9	0.06
Height [cm]	1.72 ± 0.10	1.74 ± 0.07	0.24
BMI [kg.m^−2^]	26.4 [23.5–27.8]	27.8 [24.3–28.7]	0.08
ASA-Classification	3 [3–3]	3 [3–3]	0.65

**Fig. 2:**
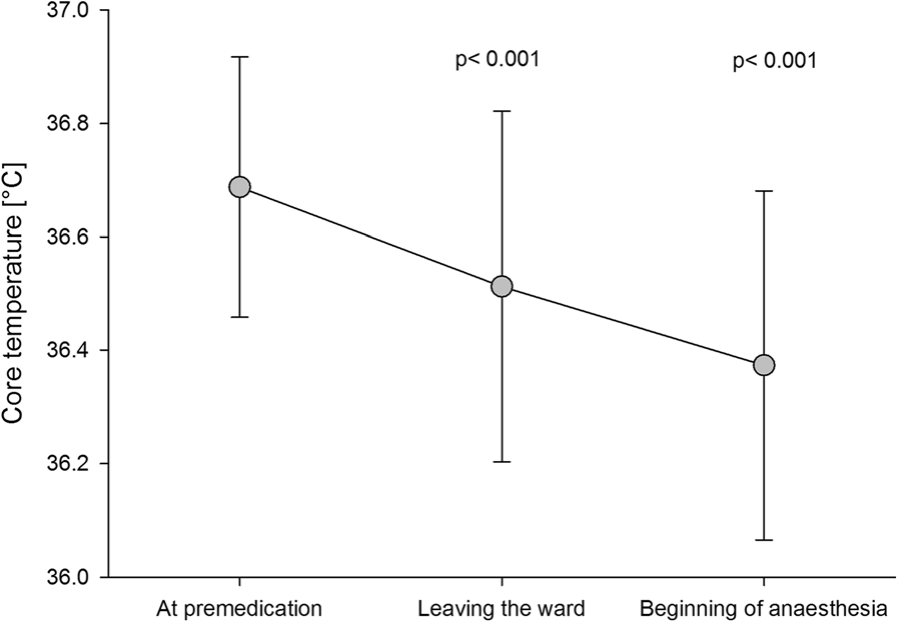
Development of core temperature after premedication. Core temperature before beginning of anaesthesia was significantly lower than core temperature at premedication and significantly lower than core temperature when leaving the ward

The first null hypothesis that the premedication with flunitrazepam does not change the core temperature was rejected. Baseline temperature of all patients was 36.7 ± 0.2 °C and dropped significantly after oral premedication with 1 mg flunitrazepam to 36.5 ± 0.3 °C when the patients were leaving the ward and to 36.4 ± 0.3 °C before induction of anaesthesia (Fig. 2).

**Fig. 3:**
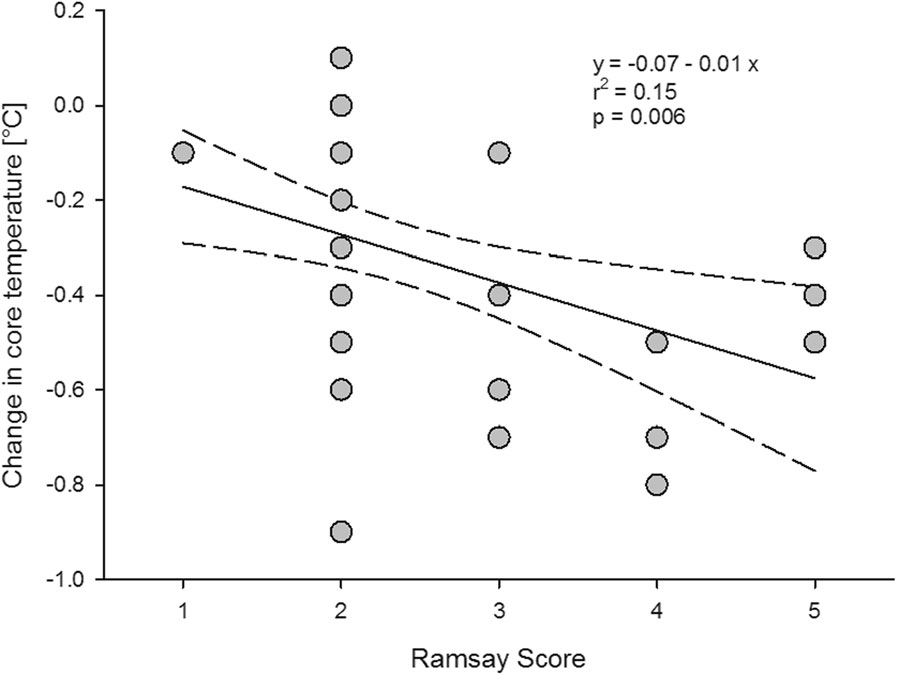
Correlation between the level of sedation and the core temperature before induction of anaesthesia. Regression line and 95% confidence intervals

The level of sedation of all patients was 2 [2–2.75] on the Ramsay scale before induction of anaesthesia. There was a clear correlation (r^2^ = 0.15) between the level of sedation and the change in core temperature between premedication and induction of anaesthesia (Fig. 3).

**Fig. 4:**
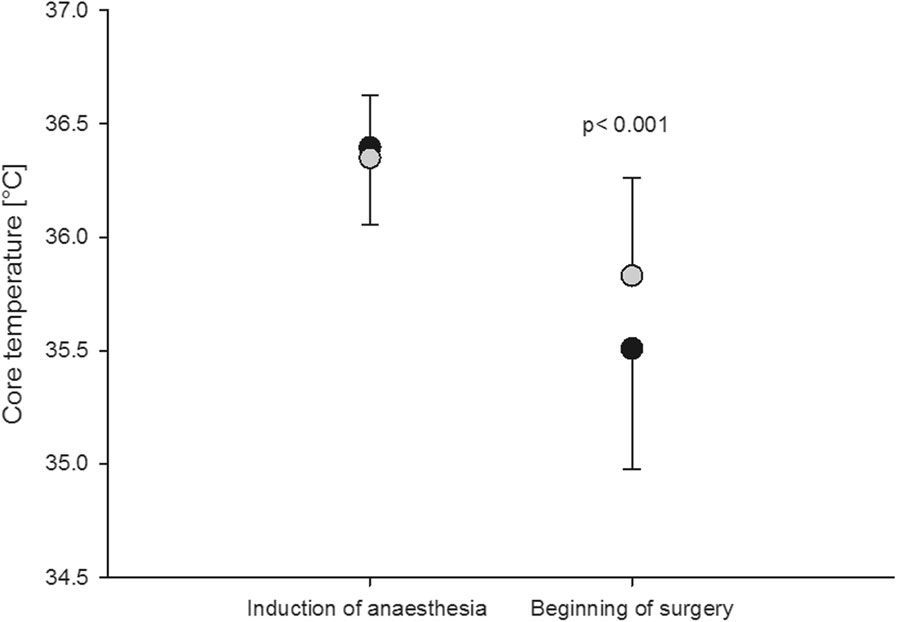
Development of core temperature after induction of anaesthesia. Core temperature dropped significantly after induction of anaesthesia in the control group (grey) compared to the prewarming group (black)

The second null hypothesis that prewarming with forced-air would not make a difference compared to no prewarming at the beginning of surgery was also rejected. The patients of the prewarming group had a significantly higher core temperature at the beginning of surgery (35.8 ± 0.4 °C) compared to patients of the control group (35.5 ± 0.5 °C, *p* = 0.027), although core temperature at induction of anaesthesia was comparable (36.4 ± 0.3 °C vs. 36.4 ± 0.3 °C, *p* = 0.611; Fig. 4). However, despite prewarming core temperature did not reach baseline level prior to premedication (36.7 ± 0.2 °C).

The incidence of perioperative hypothermia at the beginning of surgery was significantly higher (79.2%) in the control group compared to 45.8% in the prewarming group (*p* = 0.036).

## Discussion

In this study we tried to answer two questions and established two hypotheses. First, does premedication with flunitrazepam lower the core temperature significantly? Second, does prewarming have an effect on core temperature at the beginning of surgery? In this randomized controlled trial we demonstrated that premedication with flunitrazepam lowered the core temperature significantly. Further, we were able to observe that the core temperature of the patients in the prewarming group was significantly higher at the beginning of surgery compared to those of the control group. Therefore we were able to confirm both hypotheses. However, a short period of prewarming with forced-air was not able to restore the core temperature to the baseline level before premedication.

### Premedication of patients with benzodiazepines

Sedative and anxiolytic premedication is widely administered before surgery although little clinical evidence supports its use [17, 18]. In the last year routine premedication of patients with benzodiazepines has been questioned for several reasons. First, in a prospective randomized trial in patients undergoing elective surgery under general anaesthesia, premedication with lorazepam compared with placebo or no premedication failed to improve the self-reported patient experience. Even in a subgroup of the most anxious patients no significant differences were found in the global patient experience, even though anxiety of the treated patients was less compared to placebo. In contrast to these small differences induced by anxiolytic medication with a benzodiazepine there were clear disadvantages of this treatment. The time to extubation was modestly prolonged and patients had a lower rate of early cognitive recovery [17].

Second, the treatment of surgical patients with benzodiazepines is associated with postoperative delirium, especially in elderly patients [18, 19]. Postoperative delirium is a devastating complication that is clearly associated with increased mortality [18, 20, 21].

### Influence of premedication with benzodiazepines on perioperative core temperature

Until now, it is not clear if premedication with benzodiazepines increases the risk of perioperative hypothermia. The effects of benzodiazepines on perioperative core temperature have been studied with conflicting results. In a well conducted study in young and healthy volunteers Kurz et al. [11] found that even high doses of midazolam (about 40 mg in 4 h) had only moderate effects on autonomic thermoregulation. In addition, Toyota et al. [22] found no difference in core temperature after patients were premedicated with 0.04 mg.kg^− 1^ or 0.08 mg.kg^− 1^ midazolam i.m. 30 min before induction of anaesthesia. Maurice-Szamburski et al. [17] also found no difference in core temperature at induction of anaesthesia when patients received 2.5 mg Lorazepam p.o. or not.

In contrast, Gilbert et al. [23] found that core temperature of volunteers decreased about 0.3 °C after administration of 30 mg of temazepam p.o.. A similar result was obtained by Matsukawa et al. [24] in young healthy volunteers. They found that midazolam given i.m. had a clear dose dependent effect on core temperature 30 min after administration with a drop in core temperature of more than 0.5 °C when 0.075 mg.kg^− 1^ midazolam were given. In another study administration of 0.075 mg.kg^− 1^ midazolam i.m. was also associated with a drop of core temperature of 0.5 °C [12]. These results are comparable to the results of our study in which core temperature dropped 0.3 °C between administration of flunitrazepam and induction of anaesthesia. The drop in core temperature seems to be depending on the level of sedation, with the patients being more sedated having the bigger drop in core temperature [22, 24]. This effect could also be seen in our study.

Today we can only speculate about the effect of premedication with benzodiazepines on the incidence of perioperative hypothermia. In one clinical study [17], premedication with a benzodiazepine had no influence on the postoperative core temperature. However, only 50% of the patients were warmed actively and it is difficult to rule out an effect of the premedication on intraoperative and postoperative core temperature. In a second clinical study [22] premedicated patients had a smaller drop in intraoperative core temperature compared to patients without premedication. However, in both studies premedication did not lower core temperature before induction of anaesthesia as we have observed. When patients arrive in the operating room with a significant lower core temperature it seems reasonable to assume that this would lead to a lower intraoperative core temperature and a higher incidence of perioperative hypothermia. This seems especially true, if this drop in core temperature, as we have shown in our study, cannot be offset by active prewarming. This result is in contrast to the findings of Sato et al. [12] who observed that prewarming did not prevent a transient decrease in core temperature by midazolam, but increased the temperature to the control level thereafter. However, in our study active prewarming was started about 40 min after premedication and not at the time of premedication.

Active prewarming before induction of anaesthesia reduced significantly the further drop in core temperature after induction of anaesthesia and thereby the incidence of hypothermia at the beginning of surgery. Therefore we would like to underline the importance of prewarming, especially in premedicated patients.

### Strengths and weaknesses of the study

The study was conducted with a well validated method of core temperature measurement [13, 14, 25]. In contrast to many other methods of core temperature measurement, the use of a zero-heat flux thermometer allowed us to standardize the measurement and measure core temperature in awake and anaesthetized patients using the same method and the same place. Therefore we did not observe a difference in core temperature when the temperature measurement method was changed as it has been shown quite often [26, 27].

Another strength of this study is that the patients were not young and healthy as in many other studies [11, 12, 22, 24], therefore these patients are more representative for daily real life practice. We decided to conduct this study on a cohort of cardiac surgery patients, first, because these patients are usually not young and healthy. Second, these patients are premedicated with a potent benzodiazepine and third surgery with hypothermic cardio-pulmonary bypass (CPB) allowed us to create a control group of patients without prewarming (contrary to the recommendation of the national guideline [15]).

However, the study also has some weaknesses. It was a single center study with a small number of patients, but a power analysis was done and yielded satisfying results. The fact that flunitrazepam was used as premedication is not necessarily representative for daily practice. And neither weight adjustment nor BMI correlation were considered, for the dosing of the anxiolytic drug followed the standard drug dosing for cardiac surgical patients of our department. However, at least to a certain degree, these results should be comparable to other benzodiazepines.

### Open questions

To our opinion it is not clear to which extend the observed results of flunitrazepam are comparable to the effects of other benzodiazepines as midazolam. Further studies will have to clarify whether other benzodiazepines, when administered p.o. on the ward, decrease core temperature to the same extent as flunitrazepam. It remains also unclear whether the use of premedication would be associated with a higher or even lower incidence of perioperative hypothermia if patients are treated with a modern temperature management concept consisting of active prewarming and active warming during anaesthesia.

## Conclusions

Oral premedication with benzodiazepines on the ward lowered core temperature significantly at arrival in the operating room. This drop in core temperature cannot be completely offset by a short period of active prewarming.

## Abbreviations

ASA: American Society of Anesthesiology
BMI: Body Mass Index
CPB: Cardiopulmonary Bypass
